# The Ventilation Efficiency of Urban Built Intensity and Ventilation Path Identification: A Case Study of Wuhan

**DOI:** 10.3390/ijerph182111684

**Published:** 2021-11-07

**Authors:** Jie Yin, Qingming Zhan, Muhammad Tayyab, Aqeela Zahra

**Affiliations:** 1 College of Civil Engineering and Architecture, China Three Gorges University, No. 8, University Road, Xiling District, Yichang 443002, China; yinjie2014cn2014@whu.edu.cn; 2 School of Urban Design of Wuhan University, No. 8, Donghu South Road, Wuchang District, Wuhan 430072, China; qmzhan@whu.edu.cn; 3 College of Hydraulic and Environmental Engineering, China Three Gorges University, No. 8, University Road, Xiling District, Yichang 443002, China; 4 College of Economics and Management, China Three Gorges University, No. 8, University Road, Xiling District, Yichang 443002, China; 5 School of Chemistry, Chemical Engineering and Life Sciences, Wuhan University of Technology, Wuhan 430070, China; Zahra@whut.edu.cn

**Keywords:** urban built intensity index, ventilation efficiency index, ventilation path, geographic information system (GIS), computational fluid dynamics (CFD)

## Abstract

Urban ventilation is being hampered by rough surfaces in dense urban areas, and the microclimate and air quality of the urban built environment are not ideal. Identifying urban ventilation paths is helpful to save energy, reduce emissions, and improve the urban ecological environment. Wuhan is the capital city of Hubei, and it has a high urban built intensity and hot summers. Taking Wuhan city, with a size of 35 km ×50 km, as an example, the built environment was divided into grids of 100 m × 100 m and included the building density, floor area ratio, and average building height. The ventilation mechanism of the urban built intensity index has previously been explained. The decrease in building density is not the sole factor causing an increase in wind speed; the enclosure and width of the ventilation path and the height of the front building are also influential. Twelve urban built units were selected for CFD numerical simulation. The ventilation efficiency of each grid was evaluated by calculating the wind speed ratio, maximum wind speed, average wind speed, and area ratio of strong wind. The relationship between the urban built intensity index and ventilation efficiency index was established using the factor analysis method and the Pearson correlation coefficient; building density and average building height are the most critical indexes of ventilation potential. In addition, the layout of the building also has an important impact on ventilation. A suitable built environment is that in which the building density is less than 30%, the average building height is greater than 15 m, and the floor area ratio is greater than 1.5. The urban built intensity map was weighted to identify urban ventilation paths. The paper provides a quantitative reference for scientific planning and design of the urban spatial form to improve ventilation.

## 1. Introduction

In the urban climate, the intensity of a heat island can reach 12 °C under windless and minimally clouded weather conditions [1,2]. Good outdoor ventilation can help alleviate the urban heat island effect. When the air flows are at the height of pedestrians on the street, the measured temperature is 5 ℃ lower than that of the urban canopy. The heat island effect has a particularly obvious effect in Hong Kong [3].

The urban ventilation path is helpful for ventilation in the urban built environment [4,5]. In the urban built environment of Wuhan, there is fresh air in the suburbs and green island, but the air quality in the densely built environment is poor [6,7], and there is both heat and air pollution [8,9]. A ventilation path is a good and effective way to introduce fresh air located in the suburbs (green and blue places) of the city into the city center. The exhaust gas of urban pollution can be diluted and discharged with the wind, and thus, it has an important role in promoting eco-friendly circulation within the city, especially during the hot summers [10]. Opening ventilation paths in the city can reduce the air temperature; unobstructed urban ventilation paths are conducive to energy saving and emission reduction, can make the lives of urban residents more comfortable, and can reduce the energy consumption activities of urban residents due to resistance to the urban thermal environment [11]. Therefore, this approach is deemed good for the health of residents. The research into urban air ventilation is gradually developing toward multisource data integration and multiparameter quantification. We can use multisource data (such as GIS data, remote sensing image inversion data, WRF/urban microclimate simulation data, CFD simulation data, meteorological data, and urban topography, land, road, and building data) for simulation and analysis to provide recommendations in relation to decision making [12].

In terms of the quantification of the ventilation potential in the spatial form index, research mainly focuses on the quantification of multiple parameters. Adolphe uses GIS technology to propose a set of methods called morphology, which abstracts the complex urban built environment form into morphological index parameters to establish the quantitative relationship between the thermal environment, urban form, and wind [13,14], However, while these indicators focus on building morphology, they cannot be well adapted to urban planning management. Therefore, this study selects three indicators, namely, building density, floor area ratio, and building height in the controlled detailed planning, to study the ventilation value of the intensity indicators as the basis for urban planning and management. 

The urban built intensity indexes are selected as factors of the urban heat island [15]. The significant impact of urban morphology on land surface temperature was observed in different types of urban functional zones. The built intensity, represented by factors such as building density, floor area ratio, and building height, has a strong correlation with urban ventilation in Tokyo [16]. We can change the urban built intensity and optimize a building’s height, width, and depth to improve ventilation efficiency [17,18]. The urban built intensity can be used as the basis for ventilation path identification and the design standard of form optimization [19].

In the identification of the urban ventilation path, urban morphology indicators have been expanded, the number of morphological indicators increased, and layering carried out. A relationship between the morphological indicators, wind pressure, and wind speed was then established via overlay analysis, and the urban morphology design standards and optimization strategies were effectively proposed [20]. The ventilation weight of the urban built intensity is different. In this study, grid layer technology was used to perform a weighted summation of building density, floor area ratio, and building height to obtain a comprehensive evaluation result.

Zhan Qingming established a comprehensive application of remote sensing inversion (RS), geographic information spatial analysis technology (GIS), and a climatology and ecology model (WRF). Remote sensing inversion can identify the function space and compensation space in an area. GIS quantification can excavate the ventilation path of a built environment and fit it with the ventilation path analyzed by WRF meteorological simulation [21]. This study used computational fluid dynamics and geographic information systems to conduct a comprehensive analysis, focusing on the analysis of the influence mechanism of urban built intensity changes on the ventilation efficiency by CFD so as to evaluate the entire urban ventilation system with GIS. 

Urban built intensity is an important management basis for urban planning. However, there is a scarcity of research on the quantitative relationship between urban built intensity and ventilation potential [22]. There is a lack of spatial optimization technologies and standards for urban built environments [23]. Moreover, we have to verify the improvement of ventilation and heat islands by ventilation paths [24,25]. Taking Wuhan city as an example, the built environment was divided into grids and included the building density, floor area ratio, and average building height. The ventilation efficiency of each grid was evaluated by calculating the wind speed ratio, maximum wind speed, average wind speed, and area ratio of strong wind. The relationship between the urban built intensity and ventilation efficiency was analyzed so as to guide the evaluation of ventilation potential and the identification of ventilation paths in Wuhan.

## 2. Materials and Methods

### 2.1. Urban Built Intensity

The urban built intensity indexes of the urban built environment are building density (BD), floor area ratio (FAR), and average building height (ABH). They are also the controlling detailed planning indicators of urban planning. The ventilation efficiency analysis of urban built intensity indexes can be better understood and applied by planners than the roughness parameter. Buildings as three-dimensional spaces affect the ventilation environment of the city [26]. The urban built intensity index abstracts the three-dimensional building environment into a two-dimensional area to help us understand the ventilation mechanism. Field measurement, wind tunnel experiment, weather station data, remote sensing image inversion [27], weather model, computational fluid dynamics [28,29], and geographic information system research methods are comprehensively applied to study the quantitative relationship between urban built intensity and ventilation potential [30]. The equation of urban built intensity is as follows [31]:

(1) $$B\mathrm{uilding}\;D\mathrm{ensity}=\frac{\mathrm{Building}\;\mathrm{Coverage}\;\mathrm{Area}}{\mathrm{Area}\;\mathrm{of}\;\mathrm{the}\;\mathrm{Plot}\;}$$

(2) Building Average Height=Average(Building Height)

(3) $$F\mathrm{loor}\;A\mathrm{rea}\;R\mathrm{atio}=\frac{\mathrm{Gross}\;\mathrm{Floor}\;\mathrm{Area}}{\mathrm{Area}\;\mathrm{of}\;\mathrm{the}\;\mathrm{Plot}}$$

The floor area ratio, building density, and average building height are closely related. When the building density is constant, the floor area ratio is higher with the increase in the average building height; when the building average height is constant, the floor area ratio is higher with the increase in the building density. When the floor area ratio is increasing, there are two situations of wind speed changing. In a previous study, wind tunnel tests were carried out on several block plots, the building model was placed in the wind field, and correlation analysis was conducted on the average wind speed of the building plots with different building densities and floor area ratios. It was found that building density was the most important factor affecting the ventilation of the plot [32].

**Situation one:** The increase in the floor area ratio is due to the large land occupation, and the building form reduces the air velocity. However, when the building form is appropriate, for example, when the wind direction is parallel, the wind channel effect is significant, and the wind speed does not decrease but increases.

**Situation two:** The increase in floor area ratio is due to the increase in the building height, and the building form enhances the wind speed in special circumstances, because the airflow with higher wind speed at a higher height is blocked by the building and blowing to the ground. However, at the same time, the large wind shadow area also exists. The higher buildings are panel buildings and tower buildings; panel buildings form a large area of wind shadow, while the wind shadow of the tower buildings is relatively small.

### 2.2. Urban Ventilation Potential

The Wuhan urban building environment was abstracted as a two-dimensional grid (grid-scale was 100 m × 100 m) by the geographic information system (GIS). We can calculate the morphological parameters of the urban built intensity; the ventilation potential assessment of the overall built environment can be realized; and the cost of simulation can be reduced, and it is accurate, conducted in real time, and has full coverage. The wind field of each grid was simulated by computational fluid dynamics (CFD), and the ventilation efficiency of each grid was evaluated by calculating the wind speed ratio, maximum wind speed, average wind speed, and area ratio of strong wind. (1) The wind speed ratio is the ratio of the air velocity near the ground to the air velocity at the upper level, and the wind speed decreases when the air passes through the city. (2) The maximum wind speed is the highest wind speed at the height of 1.5 m in the ventilation grid, and the wind speed increases because of the physical interaction between the airflow and the building. (3) The average wind speed is the average wind speed at the height of 1.5 m in the ventilation grid, which is the overall ventilation level of the ventilation grid. (4) The area ratio of a strong wind is the ratio of the area with higher wind speed to the area of the total grid. The equation of the ventilation potential is as follows [33]:

(4) $$\alpha_{{}_{{}_{\mathrm{Wind}\;\mathrm{speed}\;\mathrm{ratio}}}}=\frac{V_{\mathrm{wind}\;\mathrm{speed}\;\mathrm{at}\;1.5m}}{V_{\mathrm{Initial}\;\mathrm{wind}\;\mathrm{speed}}}$$

(5) $$V_{\mathrm{maximum}\;\mathrm{wind}\;\mathrm{speed}}=MAX(V_{\mathrm{wind}\;\mathrm{speed}\;\mathrm{at}\;1.5m})$$

(6) $$V_{\mathrm{average}\;}=\mathrm{Average}(V_{\mathrm{wind}\;\mathrm{speed}\;\mathrm{at}\;1.5m})$$

(7) $$\beta_{\mathrm{area}\;\mathrm{ratio}\;}=\frac{{\mathrm{Area}}_{\mathrm{wind}\;\mathrm{speed}\;\mathrm{above}\;1m/s}}{{\mathrm{Area}}_{\mathrm{total}\;\mathrm{grid}}}$$

The boundary condition analysis of the ventilation environment was an important preparatory step, and it mainly consisted of the frequency statistics of wind direction and wind speed. The purpose of this paper was to obtain the dominant wind direction in summer in Wuhan city and take it as the initial boundary condition. CFD numerical simulation analysis of urban form was conducted to analyze the influence of the urban built intensity on ventilation potential. Fluent aerodynamics software was used to simulate the outdoor wind environment. The software has high accuracy in simulating the outdoor wind environment, and the ventilation statistical unit was placed in the center of the plot. According to the correlation between urban built intensity and ventilation potential indexes, the Pearson correlation coefficient method was used to calculate the weight of urban built intensity indexes’ contribution to ventilation potential. Finally, according to the weight of statistical analysis, the grid layers of BD, FAR, and ABH indexes were weighted and added to obtain a comprehensive ventilation potential evaluation map, which could be used as the basis for air path identification.

### 2.3. Urban Building Data

The data sources of this study mainly included 2010 building census data and 2004–2010 meteorological data. CFD simulation and GIS spatial analysis were used to evaluate ventilation efficiency. CFD simulation software is fluent. In this paper, fluent software was selected to simulate the typical blocks in the case city, and the spatial morphological index of the grid was quantified by ArcGIS.

The selected grid scale was 100 m × 100 m, and BD, ABH, and FAR indexes were quantified by ArcGIS. In the CFD simulation, the simulation unit scale was 300 m × 300 m (including the adjacent units of the grid); the wind data area was 100 m × 100 m (the middle 100 m × 100 m grid was taken as the research object) in the wind field simulation, and the efficiency ventilation was classified. MATLAB was used for mathematical statistics, and the quantitative relationship between the index and ventilation potential was established through spatial analysis, regression analysis, and spatial correlation analysis. Finally, the overall ventilation potential of the city was comprehensively evaluated by the overlay calculation technology of GIS. Based on the correlation degree between the urban built intensity index and ventilation efficiency, this paper classified the overall city ventilation quantitatively to identify the wind path.

**Figure 1:**
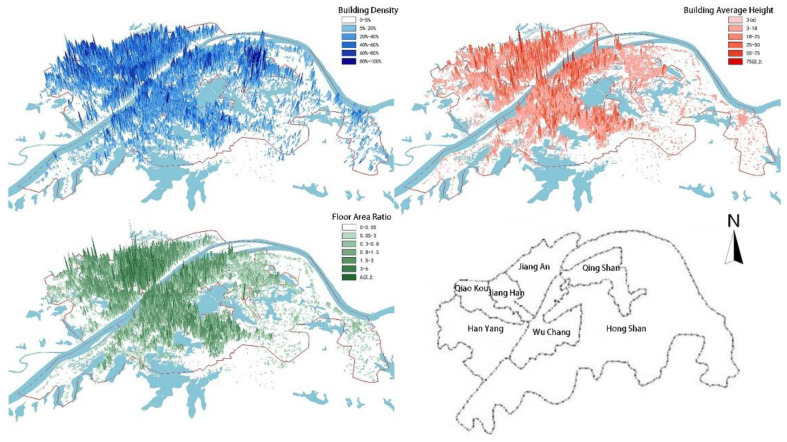
Distribution and administrative division of development intensity of the main urban area of Wuhan – building density; – average building height; – floor area ratio; – administrative division of main city area.

The spatial structure of Wuhan is a multicenter city. The distribution of the high floor area ratio in the center is uniform. In the distribution map of building density (as shown in Figure 1), the building density in the east side of the Qingshan district is the highest. The maximum building density is more than 80% because of the large number of factory buildings. The building density along the Yangtze River in the Hankou, Jiangan, and Jiangan districts is relatively high, and the building density of Donghu Lake in Hongshan district is relatively low due to the limitation on building construction. In the height distribution map (as shown in Figure 1), buildings with a height above 75 m are mainly situated along the Yangtze River in the center of the city, and the average height of buildings in the Qingshan and Hongshan districts is relatively low. In the distribution map of floor area ratio (as shown in Figure 1), the intensity of Hankou city is higher than that of Wuchang and Hanyang, and the floor area ratio of the Qingshan and Hongshan districts is generally low. The building environment of Hankou is denser than that of Wuchang and Hanyang, so the ventilation environment of Hankou is relatively poor, representing the main problem of urban ventilation.

### 2.4. Wind Direction Frequency Data

**Figure 2:**
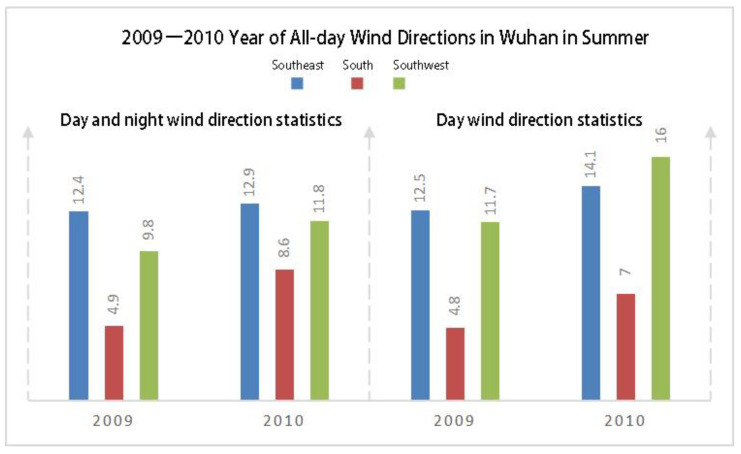
Wind frequency of an entire day in summer.

The climate data of Wuhan (including the wind data of 2009 and 2010) was based on the wind direction frequency data of summer (June, July, August, and September) and all-day wind direction frequency data. The prevailing wind directions of Wuhan in summer are southeast, south, and southwest. The frequency of the southwest direction is higher than that of the south and southeast directions. The southwest direction, with a frequency of 16%, was selected as the dominant wind direction in summer (Figure 2) and as the boundary condition for CFD simulation, GIS evaluation, and path identification.

## 3. Results

### 3.1. Ventilation Mechanism of Urban Built Intensity

There is an obvious relationship between building density, floor area ratio, and average building height. When the floor area ratio is unchanging, the higher the building density, the lower the average building height; when the average building height is unchanging, the higher the building density, the higher the floor area ratio; when the building density is unchanging, the higher the average building height, the higher the floor area ratio. The simulation condition was the south wind direction, and the wind speed was 5 m/s. The flow mode in the simulation adopted the turbulent flow mode, and the gravity acceleration was *Z* = 9.81 (m/s), while the roughness of the land surface *α* = 0.16, *Z*_0_ = 10 m.

**Figure 3:**
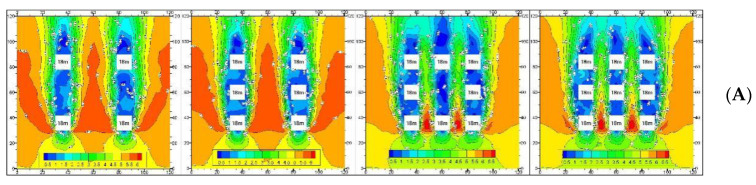

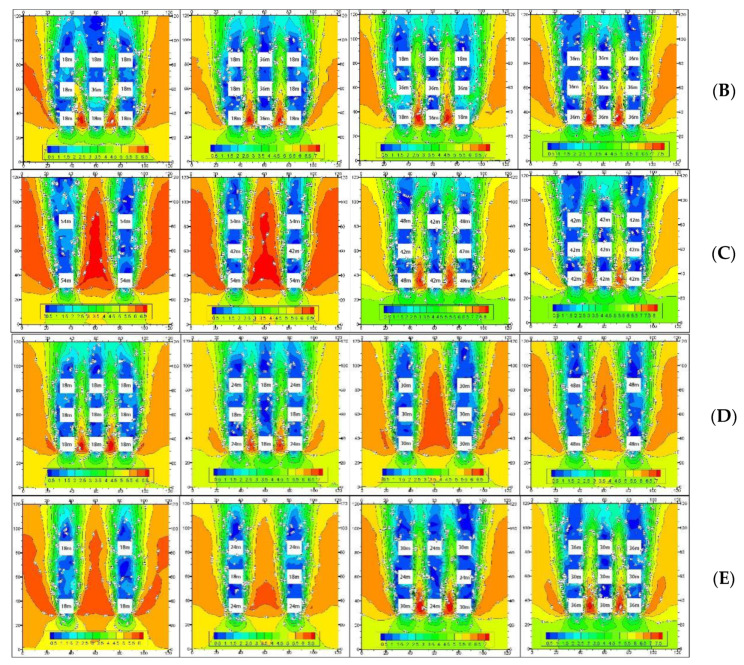
Ventilation map of building intensity.(**A**) The average building height is unchanged, the building density increases, and the floor area ratio increases; (**B**) the building density is unchanged, the average building height increases, and the floor area ratio increases; (**C**) the average building height decreases, the building density increases, and the floor area ratio increases; (**D**) the average building height increases, the building density decreases, and the floor area ratio increases; and (**E**) the average building height increases, the building density increases, and the floor area ratio increases.

Therefore, there are five situations with an increase in the building floor area ratio. The interaction mechanism between urban intensity index and ventilation potential is as follows (Figure 3):

**Situation A:** The average building height was unchanged, the building density increased, and the floor area ratio increased. The average wind speed increased. The average wind speed of case 2 (1.14 m/s) was less than that in case 1 (1.67 m/s), the building density increased, and wind speed decreased. However, the average wind speed of cases 3 and 4 was higher than that of cases 1 and 2 (Table 1), because the added buildings created a ventilation path, the wind direction was parallel to the ventilation path, and the buildings on both sides of the path increased the wind speed due to the wind drive effect.

**Situation B:** The building density was unchanged, the average building height increased, and the floor area ratio increased. The average wind speed increased. Since the building density did not change, the height of the building increased, and the building drew the high-speed wind from the upper floor to the ground, so the wind speed near the ground increased (Table 1).

**Situation C:** The average building height decreased, the building density increased, and the floor area ratio increased. The average wind speed of case 2 (1.77 m/s) was less than that of case 1 (1.78 m/s), and the average wind speed of case 4 (2.85 m/s) was less than that of case 3 (2.95 m/s) (Table 1), because the building density increased and the height of the windward building was high. The average wind speed of cases 3 and 4 was higher than that of cases 1 and 2 (Table 1), because the buildings on both sides of the path increased the wind speed.

**Situation D:** The average building height increased, the building density decreased, and the floor area ratio increased. The average wind speed of case 2 (1.77 m/s) was less than that of case 1 (2.17 m/s), and the average wind speed of case 4 (2.38 m/s) was higher than that of case 3 (1.7 7 m/s) (Table 1), because the building density decreased and the height of the windward building was higher. However, the average wind speed of cases 3 and 4 was lower than that of cases 1 and 2 (Table 1), because the width of their ventilation paths was large, and the wind speed at the wind gap was not high.

**Situation E:** The average building height increased, the building density increased, and the floor area ratio increased. The average wind speed of case 2 (1.88 m/s) was lower than that of case 1 (2.14 m/s), because the building density increased. The average wind speed of case 4 (2.76 m/s) was higher than that of case 3 (2.46 m/s) (Table 1), because the height of the windward building was high and the ventilation path was obvious. The average wind speed of cases 3 and 4 was higher than that of cases 1 and 2 (Table 1). Since the added buildings created a ventilation path, the wind direction was parallel to the ventilation path, and the height of the windward building was high.

The urban ventilation environment is closely related to the built environment. Urban built intensity indexes have different ventilation mechanisms that are related to the urban built intensity value. In addition to the urban built intensity, building layout forms are also closely related to ventilation. With the increase in the floor area ratio, the decrease in building density is not the only factor causing an increase in wind speed. When the average building height and the height of the windward building increase, the ventilation path enclosed by the building is parallel to the wind direction, and the width is small, the wind speed also increases.

### 3.2. CFD Simulation of Urban Built Unit

Twelve urban built units in Wuhan city were selected, and the unit size was 100 m * 100 m. The calculated urban built intensity indexes were building density, floor area ratio, and average building height. Each urban built intensity index was simulated for four urban built units. As an example, building density was divided into four zones, namely, building densities of 20–30%, 30–40%, 40–50%, and 50–60%. Similarly, the floor area ratio was divided into four zones, namely, floor area ratios of 0.3–0.8, 0.8–1.5, 1.5–3.0, and 3.0–6.0, and the average building height was divided into four zones, namely, average building heights of 3–18 m, 18–25 m, 25–50 m, and higher than 50 m.

The initial wind direction was southwest wind (225° from the north), and the initial wind speed was 5 m/s. The size of the simulated area was 300 m × 300 m (the grid around the urban built unit was divided into the simulation area, among which the layout form of the surrounding grid was the same). The distance between the building windward surface and the boundary in the simulated area was 1.5 times the length of the unit side, and the distance from the back wind surface to the boundary was 3 times the unit side length. The size of the grid was kept within 0.05 times the length of each coordinate axis in the simulated zoom. Here, the wind speed was 5 m/s, and the wind direction was southwest. The height of the anemometer was 10 m, the topography parameter of the meteorological station was 0.14, and the thickness of the boundary layer of the meteorological station was 270 m.

**Figure 4:**
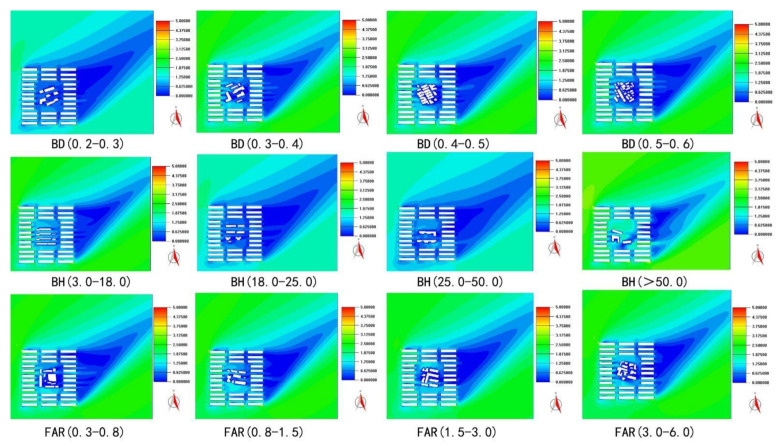
Wind speed simulation distribution of ventilation unit.

The main ventilation potential indexes were the wind speed ratio, average wind speed, maximum wind speed, and strong wind area ratio. The wind speed ratio was the ratio of the given nodes’ average wind speed at a height of 1.5 m to the background wind speed of 5 m/s. The calculation range of average wind speed and maximum wind speed was the wind field at the height of 1.5 m in the plot. The strong wind area ratio was the ratio of the area with a wind speed of more than 1 m/s to the total area of the urban built unit at the height of 1.5 m. To compare the correlation between the ventilation potential and urban built intensity of each case, four interval cases of 0.2–0.6 were adopted for building density comparison, four interval cases of 3–50 m were adopted for average building height comparison, and four interval cases of 0.3–6.0 were adopted for floor area ratio comparison. The correlation between building density and ventilation potential was the highest, followed by the average building height, and the correlation between floor area ratio and ventilation potential was not obvious, as shown in Figure 4 and Table 2. The variable urban built intensity indexes gradually increased, and the other two urban built intensity indexes relied on the actual built environment.

### 3.3. Ventilation Potential and Urban Built Intensity

**Figure 5:**
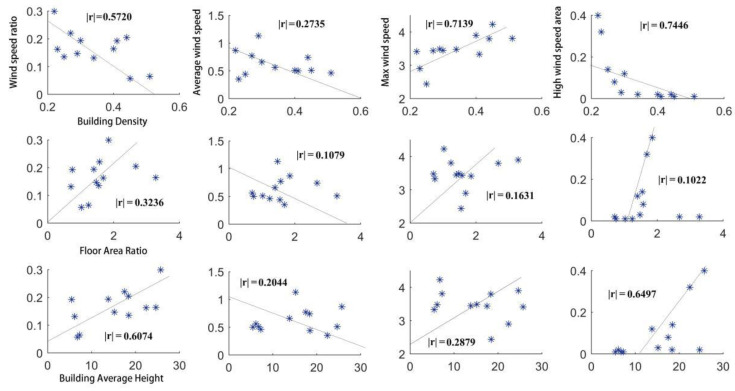
Scatter plot of correlation between wind ventilation potential and development intensity.

Via statistical analysis of the correlation between the ventilation potential and urban built intensity (Figure 5), it was found that building density and ventilation potential were clearly correlated, but average wind speed was weakly correlated with building density; the correlated value between the floor area ratio and ventilation potential was less than 0.5; the average building height was generally correlated with ventilation potential, but the average and maximum wind speed was weakly correlated with average building height. The correlation between the four ventilation potential variables and the urban built intensity was inconsistent. To overcome the problem of too many indexes and inconsistent correlation of the indexes, as well as improving the accuracy and rationality of the analysis results, the factor analysis method was used to reduce the dimensions of the four ventilation potential variables, and the correlation between the comprehensive score after dimension reduction and the urban built intensity indexes was analyzed by weight.

Two common factors (F1 and F2) were obtained by selecting the common factor whose eigenvalue was greater than 1 or the cumulative contribution rate of common factor variance greater than 80%, and the cumulative contribution rate of common factor variance reached 80.79%. The percentage of each factor’s variance contribution rate in the sum of the factor’s variance contribution rate was taken as the weight, and the weighted sum of each factor’s score was obtained. This allowed us to obtain a comprehensive score of ventilation potential. The higher the score, the stronger the ventilation potential.

The factor score coefficient matrix was obtained by SAS software. The ventilation potential value *X_j_* of each ventilation unit case was multiplied (Table 2, Equation (8)), the common factor scores F1 and F2 were calculated, and the percentage of the extracted variance contribution rate of each factor was taken as the weight in the sum of the factor variance contribution rate *ε_i_* (Table 3) and according to the factor score coefficient *W_ij_* (Table 4). These factors were then summed with the common factor scores, resulting in the comprehensive scores of ventilation potential (Table 5, Equation (9)). The equation is as follows [34]:

(8) $$F_{i}=\sum_{j=1}^{12}\left(w_{\mathrm{ij}}\times X_{j}\right)$$

(9) $$F=\frac{\sum_{i=1}^{2}\varepsilon_{i}\times F_{i}}{\sum_{i=1}^{2}\varepsilon_{i}}$$

**Figure 6:**
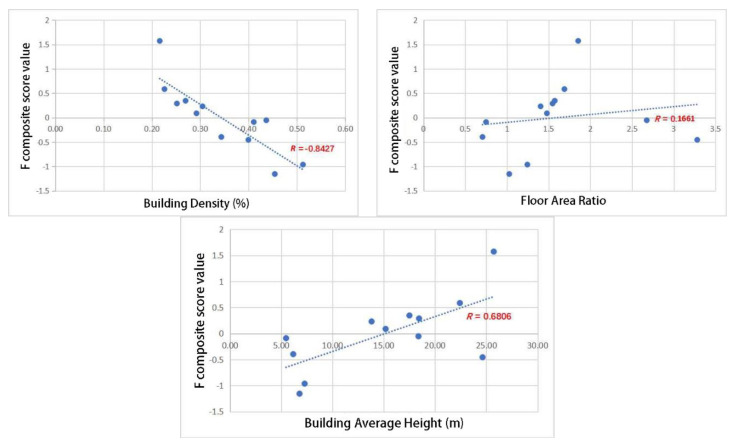
Scattered point map of the correlation degree between the comprehensive score of wind ventilation potential and development strength.

The correlation degree between the urban built intensity indexes (building density, floor area ratio, and average building height) and the ventilation potential comprehensive score was calculated using the Pearson correlation coefficient method. The calculation results (Figure 6) were as follows:

(1) The comprehensive score for ventilation potential and building density showed a strong correlation, and the correlation coefficient was −0.8427. When the building density was lower than 30%, the comprehensive score of ventilation potential was positive. (2) The comprehensive score for ventilation potential and floor area ratio showed a weak correlation, and the correlation coefficient was 0.1661. When the floor area ratio was more than 1.5, the comprehensive score of ventilation potential was positive. (3) The comprehensive score for the average height and ventilation potential showed a strong correlation, and the correlation coefficient was 0.6806. When the average height of the building exceeded 15 m, the comprehensive score of ventilation potential in most cases was positive.

According to the correlation coefficient, the weight of the influence of each urban built intensity index on ventilation potential was calculated. The weight of building density accounted for 50%, while the weights of the floor area ratio and average building height were 10% and 40%, respectively.

## 4. Discussion

In the current study, ventilation paths were detected according to the ventilation mechanism of the urban built intensity. The built environment of Wuhan city center was abstracted into grid units of 100 m × 100 m. The three indexes of building density, floor area ratio, and average building height were calculated, making up for the deficiency of single-factor evaluation and realizing the overall ventilation potential of Wuhan city center. Based on CFD numerical simulation, the wind speed ratio, average wind speed, maximum wind speed, and strong wind area ratio were calculated. The strength of the correlation between urban built intensity indexes and the ventilation potential variable was calculated using factor analysis and the Pearson correlation coefficient method. On this basis, the urban built intensity grid layer was reclassified. Using the GIS grid calculator, the urban built intensity indexes were weighted and summed, and the comprehensive evaluation map of ventilation potential was calculated.

The grid data of building density in 100 m × 100 m units of Wuhan city were classified by GIS. Because the building density was negatively related to the ventilation environment, the building density was defined as 6, 5, 4, 3, 2, and 1, and the floor area ratio and average building height were positively correlated with ventilation potential. Then, according to the weight of the relationship between the urban built intensity and ventilation environment, the ventilation environment evaluation map of Wuhan city was obtained using the grid calculator. The larger the ventilation environment value, the better the ventilation, and the values of the ventilation environment are 1–3, 3–5, and 5–6.

**Figure 7:**
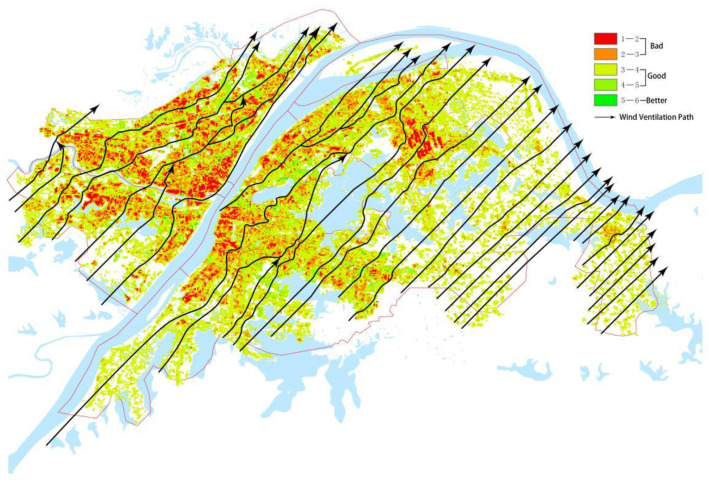
Identification map of the ventilation path in the main urban area of Wuhan city.

We took the southwest wind of Wuhan as the background wind and connected the grids with good and improved ventilation levels. The streamline conformed to the southwest-leading wind direction. Path identification was carried out, passing through the units with lower ventilation levels was avoided, and the main urban ventilation path of Wuhan was obtained (Figure 7). The ventilation potential of Qingshan district is low, because its building density is higher than 80%. The ventilation of Hankou district is relatively bad, because its building density is large, its layout is concentrated, and there is no ventilation path.

The urban built intensity determines the ventilation efficiency of the ventilation path [35,36]. As a morphological characteristic of the built environment, the urban built intensity has an obvious correlation with ventilation potential [37,38]. The characteristics have significance for urban planning. The urban built intensity determines the layout of the ventilation path. For example, there is a large difference in ventilation levels between densely built areas and open-space areas. The analysis of built environment conditions can determine the ventilation level of the city and identify the urban ventilation path. In the identification of the ventilation path in the main urban area of Wuhan city, it was found that the density of the ventilation path in the old urban area of Hankou and Wuchang was low, the path line was curved, and the ventilation environment was not ideal. The ventilation paths in other areas were straight, and the line density was higher than that in the old urban area (Figure 7).

## 5. Conclusions

Urban ventilation research has significance for urban planning [39,40]. Traditional urban planning lacks the aspect of climate research, which plays an important role in the realization of urban sustainable development and in solving urban environmental problems. Urban built intensity reflects the three-dimensional characteristics of the urban built environment and is an important index of urban planning and building management. The mechanism of urban built intensity in the ventilation potential is different. It is of great significance to identify the ventilation path for the reasonable guidance of urban planning.

Building density and ventilation potential showed an obvious correlation, floor area ratio showed a weak correlation with ventilation potential, and building height had a certain degree of ventilation potential promotion. This is because building density is not the sole factor causing an increase in wind speed. When the average building height and the height of the windward building increase, the ventilation path enclosed by the building is parallel to the wind direction, and the width is small, the wind speed also increases. A suitable built environment is that in which the building density is less than 30%, the average building height is greater than 15m, and the floor area ratio is greater than 1.5.

It is necessary to identify the existing ventilation paths in the city, as well as to protect and control such ventilation paths. We can reduce the building density to below 30%, appropriately increase the building height to 15 m or more, and increase the height of landmark buildings in densely built areas. If the street is parallel to the prevailing wind direction, the street wall can be encouraged, openings can be reduced, and the street width can be appropriately reduced to form ventilation channels and increase the height of the buildings on the windward side. Thus, a ventilation system can be formed by connecting ventilation paths in series. 

The building environment of Hankou is more dense than that of Wuchang and Hanyang, so the ventilation environment of Hankou is relatively poor, representing the main problem of urban ventilation. Based on the influence of urban built intensity on the ventilation environment, this study demonstrates the correlation strength between the building density, floor area ratio, average height of buildings, and ventilation potential, improves the accuracy of ventilation path identification, and provides a reference for urban planners and decision makers.

## Figures and Tables

**Table 1:** Wind speed of the development intensity index.

Situation	Case	Development Intensity			Wind Speed (m/s)			
		ABH (M)	BD (%)	FAR	MAX	MIN	Average	Standard Deviation
(A)	1	18	16	0.96	5.32	0.35	1.67	1.03
	2	18	24	1.44	5.83	0.15	1.14	0.77
	3	18	32	1.92	6.98	0.09	2.06	1.57
	4	18	36	2.16	6.93	0.18	2.12	1.62
(B)	1	20	36	2.4	6.78	0.07	2.2	1.58
	2	24	36	2.88	7.17	0.12	2.38	1.84
	3	28	36	3.36	7.22	0.13	2.61	1.74
	4	36	36	4.32	7.78	0.14	2.82	2.04
(C)	1	54	16	2.88	6.59	0.17	1.78	1.36
	2	50	24	4	6.69	0.19	1.77	1.23
	3	45	32	4.8	8.06	0.17	2.95	2.12
	4	42	36	5.04	7.80	0.14	2.85	2.13
(D)	1	18	36	2.16	6.92	0.16	2.17	1.63
	2	21	32	2.24	7.11	0.16	2.36	1.63
	3	30	24	2.40	5.85	0.30	1.77	1.25
	4	48	16	2.56	6.59	0.14	2.38	1.84
(E)	1	18	16	0.96	5.93	0.10	2.14	1.53
	2	22	24	1.76	6.17	0.12	1.88	1.46
	3	27	32	2.88	7.48	0.08	2.46	1.88
	4	32	36	3.92	7.67	0.09	2.76	2.02

**Table 2:** Ventilation of 12 urban built units.

Case	BD	FAR	ABH	Wind Speed (m/s)			
				Wind Speed Ratio	Average	Max	Strong Area Ratio (%)
BD (0.2–0.3)	0.29	1.48	15.18	0.15	1.13	3.49	0.026
BD (0.3–0.4)	0.34	0.71	6.17	0.13	0.56	3.48	0.018
BD (0.4–0.5)	0.45	1.03	6.79	0.06	0.51	4.23	0.010
BD (0.5–0.6)	0.51	1.24	7.29	0.07	0.46	3.81	0.005
ABH (3–18 m)	0.27	1.57	17.50	0.22	0.77	3.44	0.076
ABH (18–25 m)	0.25	1.55	18.44	0.14	0.44	2.44	0.142
ABH (25–50 m)	0.23	1.69	22.42	0.16	0.35	2.90	0.316
ABH (>50 m)	0.22	1.85	25.73	0.30	0.87	3.41	0.404
FAR (0.3–0.8)	0.41	0.75	5.48	0.19	0.50	3.33	0.013
FAR (0.8–1.5)	0.31	1.40	13.82	0.19	0.66	3.45	0.121
FAR (1.5–3.0)	0.44	2.68	18.39	0.21	0.74	3.80	0.023
FAR (3.0–6.0)	0.40	3.28	24.64	0.16	0.51	3.90	0.015

**Table 3:** Variance contribution rate (*ε_i_*).

Ventilation Potential Variable	Variance Contribution Rate (%)	Cumulative Variance Contribution Rate (%)
Wind speed ratio	48.446	48.446
Average wind speed	32.349	80.795
Maximum wind speed	12.076	92.870
Strong wind area ratio	7.130	100.000

**Table 4:** Common factor score coefficient matrix.

Ventilation Potential Variable	F1	F2
Wind speed ratio	0.243	0.453
Average wind speed	−0.194	0.664
Maximum wind speed	−0.509	0.243
Strong wind area ratio	0.472	0.067

**Table 5:** Common factor scores and comprehensive scores.

Case	F1 (Common Factor 1)	F2 (Common Factor 2)	F (Comprehensive Score)
BD (0.2–0.3)	−0.77215	1.37774	0.088631
BD (0.3–0.4)	−0.35386	−0.45978	−0.39627
BD (0.4–0.5)	−1.43698	−0.73409	−1.15555
BD (0.5–0.6)	−0.90534	−1.04852	−0.96267
BH (3–18 m)	0.04791	0.79279	0.346148
BH (18–25 m)	1.33318	−1.27336	0.289564
BH (25–50 m)	1.65968	−1.02434	0.585042
BH (>50 m)	1.43338	1.78139	1.572718
FAR (0.3–0.8)	0.05256	−0.3027	−0.08968
FAR (0.8–1.5)	0.18105	0.30656	0.231302
FAR (1.5–3.0)	−0.59164	0.75217	−0.0536
FAR (3.0–6.0)	−0.64779	−0.16785	−0.45563

## Data Availability

The data used to support the findings of this study are available from the corresponding author upon request.

## References

[1] Oke T.R. (1981). Canyon geometry and the nocturnal urban heat island: Comparison of scale model and field observations. J. Clim..

[2] Oke T.R. (1982). The energetic basis of the urban heat island. Q. J. R. Meteorol. Soc..

[3] Man S.W., Nichol J.E., Pui H.T., Wang J.Z. (2010). A simple method for designation of urban ventilation corridors and its application to urban heat island analysis. Build. Environ..

[4] Hsieh C.-M., Huang H.-C. (2016). Mitigating urban heat islands: A method to identify potential wind corridor for cooling and ventilation. Comput. Environ. Urban Syst..

[5] Gál T., Unger J. (2009). Detection of ventilation paths using high-resolution roughness parameter mapping in a large urban area. Build. Environ..

[6] Lan Y., Zhan Q. (2017). How do urban buildings impact summer air temperature? The effects of building configurations in space and time. Build. Environ..

[7] Wang Y.S., Zhan Q.M., Ouyang W.L. (2019). How to quantify the relationship between spatial distribution of urban waterbodies and land surface temperature?. Sci. Total Environ..

[8] Zhang L., Zhan Q., Lan Y. (2018). Effects of the tree distribution and species on outdoor environment conditions in a hot summer and cold winter zone: A case study in Wuhan residential quarters. Build. Environ..

[9] Liu H., Zhan Q., Gao S., Yang C. (2019). Seasonal Variation of the Spatially Non-Stationary Association Between Land Surface Temperature and Urban Landscape. Remote Sens..

[10] Ghiaus C., Allard F., Santamouris M., Georgakis C., Nicol F. (2006). Urban environment influence on natural ventilation potential. Build. Environ..

[11] Oke T. (1973). City size and the urban heat island. Atmos. Environ..

[12] Ng E., Yuan C., Chen L., Ren C., Fung J.C. (2011). Improving the wind environment in high-density cities by understanding urban morphology and surface roughness: A study in Hong Kong. Landsc. Urban Plan..

[13] Adolphe L. Modeling the link between built environment and urban climate: Towards simplified indicators of the city environment. Proceedings of the Seventh International IBPSA Conference.

[14] Adolphe L. (2001). A Simplified Model of Urban Morphology: Application to an Analysis of the Environmental Performance of Cities. Environ. Plan. B Plan. Des..

[15] Gao S.H., Zhan Q., Yang C., Liu H. (2020). The Diversified Impacts of Urban Morphology on Land Surface Temperature among Urban Functional Zones. Int. J. Environ. Res. Public Health.

[16] Lim J., Ooka R. (2014). Correlation analysis of urban morphological parameters using GIS data of Tokyo: Parameterization of urban ventilation potential in high-density urban area Part 1. J. Environ. Eng..

[17] Lim J., Ooka R. (2021). A CFD-Based Optimization of Building Configuration for Urban Ventilation Potential. Energies.

[18] Wu Y., Zhan Q., Quan S.J. (2021). A robust metamodel-based optimization design method for improving pedestrian wind comfort in an infill development project. Sustain. Cities Soc..

[19] Wu C. Modelling the Temporal and Spatial Relationship among Air Quality, Urban Morphology, and Urban Ventilation. https://www.researchgate.net/publication/349274308.

[20] Hsie T.S., Ward I.C. A GIS-based Method for Determining Natural Ventilation Potentials and Urban Morphology. Proceedings of the 23rd Conference on Passive and Low Energy Architecture.

[21] Zhan Q., Fan Y., Xiao Y., Ouyang W., Lan Y., Jin Z., Yin J., Zhang L. (2018). Sustainable Strategy: Comprehensive Computational Approach for Wind Path Planning in Dense Urban Area. Int. Rev. Spat. Plan. Sustain. Dev..

[22] Bady M., Kato S., Takahashi T., Huang H. (2011). An experimental investigation of the wind environment and air quality within a densely populated urban street canyon. J. Wind Eng. Ind. Aerodyn..

[23] Ng E. (2009). Policies and technical guidelines for urban planning of high-density cities—air ventilation assessment (AVA) of Hong Kong. Build. Environ..

[24] Qiao Z., Xu X., Wu F., Luo W., Wang F., Liu L., Sun Z. (2017). Urban ventilation network model: A case study of the core zone of capital function in Beijing metropolitan area. J. Clean. Prod..

[25] Gao Y., Yao R., Li B., Turkbeyler E., Luo Q., Short C.A. (2012). Field studies on the effect of built forms on urban wind environments. Renew. Energy.

[26] Zhou Y., Shi T., Hu Y., Gao C., Liu M., Fu S., Wang S. (2011). Urban green space planning based on computational fluid dynamics model and landscape ecology principle: A case study of Liaoyang City, Northeast China. Chin. Geogr. Sci..

[27] Lo C.P., Quattrochi D.A., Luvall J. (1997). Application of high-resolution thermal infrared remote sensing and GIS to assess the urban heat island effect. Int. J. Remote Sens..

[28] Hang J., Li Y., Sandberg M., Claesson L. (2010). Wind conditions and ventilation in high-rise long street models. Build. Environ..

[29] Yuan C., Ng E.Y.Y. (2012). Building porosity for better urban ventilation in high-density cities—A computational parametric study. Build. Environ..

[30] Guo F., Zhang H.G., Fan Y., Zhu P.S., Wang S.Y., Lu X.D., Jin Y. (2018). Detection and evaluation of a ventilation path in a moun-tainous city for a sea breeze: The case of Dalian. Build. Environ..

[31] (2014). Corporate Finance & Accounting, Financial Ratios. https://www.investopedia.com/terms/f/floor-area-ratio.asp.

[32] Kubota T., Miura M., Tominaga Y., Mochida A. (2008). Wind tunnel tests on the relationship between building density and pedestrian-level wind velocity: Development of guidelines for realizing acceptable wind environment in residential neighborhoods. Build. Environ..

[33] Liu Z.X., Han J., Zhou J., Zhang C., Zhang G. (2015). Study on Evaluation of District Wind Environment Based on Wind Velocity Ratio and Air Age. Archit. Technol..

[34] Yan C.L., Liang L.K., Liu X.J., Wang W.J. (2014). Study of the Urban Tourism Competitiveness Based on Factor Analysis: A Case of 30 Cities in Central Plains Economic Zone. Areal Res. Dev..

[35] Ho Y.-K., Liu C.-H. (2017). Street-Level Ventilation in Hypothetical Urban Areas. Atmosphere.

[36] Biehl J., Paas B., Klemm O. (2021). Ventilation of a Mid-Size City under Stable Boundary Layer Conditions: A Simulation Using the LES Model PALM. Atmosphere.

[37] Saber E., Chaer I., Gillich A., Ekpeti B. (2021). Review of Intelligent Control Systems for Natural Ventilation as Passive Cooling Strategy for UK Buildings and Similar Climatic Conditions. Energies.

[38] Sakiyama N.R.M., Frick J., Bejat T., Garrecht H. (2021). Using CFD to Evaluate Natural Ventilation through a 3D Parametric Modeling Approach. Energies.

[39] Li J., Peng Y., Ji H., Hu Y., Ding W. (2019). A Wind Tunnel Study on the Correlation between Urban Space Quantification and Pedestrian–Level Ventilation. Atmosphere.

[40] Peng Y., Gao Z., Buccolieri R., Ding W. (2019). An Investigation of the Quantitative Correlation between Urban Morphology Parameters and Outdoor Ventilation Efficiency Indices. Atmosphere.

